# A new statistical model for binge drinking pattern classification in college-student populations

**DOI:** 10.3389/fpsyg.2023.1134118

**Published:** 2023-07-17

**Authors:** Judith André, Momar Diouf, Margaret P. Martinetti, Olivia Ortelli, Fabien Gierski, Frederic Fürst, Olivier Pierrefiche, Mickael Naassila

**Affiliations:** ^1^INSERM UMR 1247, Groupe de Recherche sur l’alcool et les Pharmacodépendances, GRAP, Université Picardie Jules Verne, Amiens, France; ^2^Biostatistics Unit, Clinical Research Department, Amiens-Picardie University Hospital, Amiens, France; ^3^Department of Psychology, The College of New Jersey, Ewing, NJ, United States; ^4^Cognition, Health, Society Laboratory (C2S – EA 6291), University of Reims Champagne Ardenne (URCA), Reims, France; ^5^Fédération Hospitalo-Universitaire A2M2P, Améliore le Pronostic des Troubles Addictifs et Mentaux par une Médecine Personnalisée, Paris, France; ^6^GDR CNRS 3557 Psychiatrie-Addictions, Institut de Psychiatrie, Paris, France; ^7^Laboratoire MIS (Modélisation, Information et Système) UR 4290, Université Picardie Jules Verne, Amiens, France

**Keywords:** alcohol, binge drinking, high-intensity binge drinking, college students, drunkenness, classification tool, gender

## Abstract

**Background:**

Binge drinking (BD) among students is a frequent alcohol consumption pattern that produces adverse consequences. A widely discussed difficulty in the scientific community is defining and characterizing BD patterns. This study aimed to find homogenous drinking groups and then provide a new tool, based on a model that includes several key factors of BD, to assess the severity of BD regardless of the individual’s gender.

**Methods:**

Using the learning sample (N1 = 1,271), a *K*-means clustering algorithm and a partial proportional odds model (PPOM) were used to isolate drinking and behavioral key factors, create homogenous groups of drinkers, and estimate the probability of belonging to these groups. Robustness of our findings were evaluated with Two validations samples (N2 = 2,310, N3 = 120) of French university students (aged 18–25 years) were anonymously investigated via demographic and alcohol consumption questionnaires (AUDIT, AUQ, Alcohol Purchase Task for behavioral economic indices).

**Results:**

The *K*-means revealed four homogeneous groups, based on drinking profiles: low-risk, hazardous, binge, and high-intensity BD. The PPOM generated the probability of each participant, self-identified as either male or female, to belong to one of these groups. Our results were confirmed in two validation samples, and we observed differences between the 4 drinking groups in terms of consumption consequences and behavioral economic demand indices.

**Conclusion:**

Our model reveals a progressive severity in the drinking pattern and its consequences and may better characterize binge drinking among university student samples. This model provides a new tool for assessing the severity of binge drinking and illustrates that frequency of drinking behavior and particularly drunkenness are central features of a binge drinking model.

## 1. Introduction

Binge drinking (BD) is a major public health problem that produces several harmful consequences (Rolland et al., 2017). As the most prevalent drinking habit among Western youth (Dormal et al., 2019; Maurage et al., 2020), binge drinking increases injuries and health risks, leading to brain and cognitive alterations (Squeglia et al., 2011, 2012; Petit et al., 2014). Beyond immediate health risks, BD produces long-lasting brain and psychological impacts, suggesting that this drinking pattern may be a first step toward drug disorders in the long term (Patrick et al., 2019; Tavolacci et al., 2019; Jaeger and Oshman, 2021). Despite this impact on public health, the research community is overwhelmed with a diversity of definitions and denominations of BD (Rolland et al., 2017). Irrespective of cultural influences on the patterns of consumption, such diversity in the definition of BD leads to high variability among the markers of prevalence (Varela-Mato et al., 2012; Mahmood et al., 2016). Differences in such definitions affect categorizations of BD both within and across studies, and these differences could have a profound impact on both the results and implications of the studies.

Globally, two main definitional streams have emerged among the proposed criteria for BD. The first concerns *quantity or quantity-frequency* based definitions (Wechsler and Nelson, 2001; Rolland et al., 2017). These definitions define BD with respect to quantity of alcohol consumed, blood alcohol concentration (BAC), and/or standard alcohol dose measures (Maurage et al., 2020) (i.e., the WHO definition of ≥60 g of ethanol per occasion and the NIAAA definition of ≥56 g for women and ≥ 70 g drinks for men in a 2-h interval and blood alcohol concentration ≥ 0.8 g/L). The strictly *quantity-based definitions* have two main drawbacks, despite their ease of use and prevalence in epidemiological studies. First, accurate estimates of alcohol consumption are difficult to achieve for several reasons: (i) the amount of alcohol in a “standard drink” varies between countries (e.g., 10 g in France, 20 g in Austria, 14 g in US, and 8 g in UK); (ii) young adults frequently consume non-standardized drinks (plastic cups, etc.) (Morrell et al., 2021) in private dwellings (e.g., at home) or in a public place (e.g., public park) (Labhart et al., 2013) and (iii) the number of standard doses consumed does not take into account the consumer’s physical characteristics (tolerance, sex and body mass index) that affect BAC. Second, the use of “cut-offs,” or delineating participant groups according to the number of drinks consumed (e.g., fewer than 5/4 drinks for men/women as “non-binge” drinking), generates erroneous dichotomization and inaccurate labeling (i.e., broad categorization into a group) (DeCoster et al., 2009; Pearson et al., 2016). The use of cut-offs underestimates real consumption and mitigates actual consequences. As a dichotomous variable, BD has little predictive value for public health impact whereas a clear dose effect links BD frequency, intensity, and many negative health consequences, including mortality (Donat et al., 2021). The addition of a frequency dimension to quantity (i.e., *quantity-frequency*) improves the drinking assessment, as frequency is highly related to severity of drinking consequences (Williams et al., 1994; Greenfield, 2000). For example, the Timeline Follow Back (TLFB) (Collins et al., 2008) has been used to track BD episodes during a period of 7 days to 12 months. The degree to which a given quantity of consumption occurs within that time period provides an important temporal dimension (Williams et al., 1994; Greenfield, 2000).

The second stream of BD definitions concerns *behavioral patterns* (Townshend and Duka, 2002), or more subjective behavioral drinking “phenotypes,” such as frequency of drunkenness and hangovers. Such definitions include the adaptations of known questionnaires to rank consumption (Tuunanen et al., 2007; Olthuis et al., 2011; Cortés-Tomás et al., 2016). For example, the AUDIT questionnaire is sometimes used to assess the severity of BD (Motos-Sellés et al., 2020), although it remains incomplete for several reasons (Shakeshaft et al., 1998; Tuunanen et al., 2007; Mota et al., 2010; Olthuis et al., 2011; Cortés-Tomás et al., 2016; Motos-Sellés et al., 2020). In particular, the AUDIT questionnaire does not assess the number of drunkenness episodes or the speed of drinking, which are essential characteristics of BD behavior (Maurage et al., 2020). In addition, cut-offs for AUDIT total scores require careful adjustment to remain valid in college populations (Olthuis et al., 2011). To deal with these limitations, other behavior-based models of BD have been developed to include physiological and subjective intoxication criteria such as *drunkenness* and *frequency of drunkenness*. For example, the BD score proposed by Townshend and Duka (2002) includes a sum of *consumption speed*, *number of drunkenness episodes,* and *percentage of drunkenness episodes out of 10 drinking occasions*. Because of their subjectivity, drunkenness and frequency of drunkenness are recognized as both highly predictive of social consequences and symptoms of alcohol dependence and alcohol-related harm, and therefore, may be more valid than the amount of alcohol by subject experience, biological factors and demographic criteria (Midanik, 1999; Paljärvi et al., 2012; Sznitman et al., 2017). Speed of drinking, also included in the “BD score,” is another index of severity (López-Caneda et al., 2013; Poulton et al., 2016), increasing more rapidly in high-risk drinking (Groefsema and Kuntsche, 2019). Despite these advantages of the BD score, its utility is hampered by the percentile method used to categorize groups of participants: The lowest third is said to constitute “social drinkers” and the upper third is considered “binge drinkers,” but the intermediate percentile remains unclear and designated “intermediate.” Thus, these cutoffs will necessarily vary depending on the distribution of consumption in a given sample (Townshend and Duka, 2002).

In addition to wide variability among BD definitions, there is also variability in the inclusion of gender in these definitions. Indeed, some studies use gender-specific measures (e.g., 5+ and 4+ US standard drinks, or 70 g+/56 g+, respectively for men and women) (Wechsler and Nelson, 2001), whereas others do not (e.g., the AUDIT uses 60 g of ethanol) (Saunders et al., 1993; Babor et al., 1994; Graham et al., 1998; Wilsnack et al., 2018). Thus, lowering a threshold to define BD among women may increase the prevalence of this behavior among women (Chavez et al., 2012). Even though physiological factors, such as drunkenness, depend on the metabolism of alcohol in accordance to sex, no study to date has been able to quantify binge drinking in a manner that is valid regardless of gender.

This lack of standardization and consensus in BD definitions illustrates the variability that makes it difficult to compare findings across studies (Cortés-Tomás et al., 2016). This methodological difficulty has become a barrier to advancing public health, given the pervasiveness of BD in the population and its spectrum of consequences (Rolland et al., 2017; Maurage et al., 2020). Taken together, the adverse consequences of BD, the lack of clarity regarding BD as a mode of consumption and resulting obfuscation of research findings justify the need for a novel instrument to define and detect BD (Cortés-Tomás et al., 2016).

Therefore, the current study had three research aims: (1) starting with recognized BD criteria, develop a better tool for characterizing BD behavior that takes into account behavioral, quantitative and physiological consumption features and that defines homogeneous groups keeping all individuals from the whole population (2) with this tool, provide a stable, specific, and reproducible way to investigate other BD-related questions, especially those regarding severity and gender variability; and (3) further characterize BD groups using alcohol-related consequences and behavioral economic indices (e.g., demand intensity and breakpoint).

## 2. Methods

### 2.1. Design

#### 2.1.1. Participants

The learning sample and validation samples were recruited from three French universities (Rennes, Amiens and Reims) with different methods of recruitment.

##### 2.1.1.1. Learning sample

An online anonymous survey was distributed to all students from the University of Rennes. In total, 29,000 students were invited to complete the questionnaire via their personal university email address. A total of 1,870 students which consisted primarily of Caucasian responded to the questionnaire. Criteria of inclusion were age between 18 and 25 and drinking 5 or more drinks (50 g) per week. This population was already described in a previous study (Rolland et al., 2017). For the current study, we excluded participants with missing data, leading to a final sample of 1,277 participants (77.3% female). Students were able to continue with the survey only if they stated that they do consent to participate by ticking the consent button after reading the consent form (purpose of research, participation, procedure, confidentiality, and researcher’s contact information). With respect to the students’ academic programs, 39% reported law, economy, management, or human sciences; 28% were pursuing health studies; and 33% were in the field of sciences, engineering and technologies.

##### 2.1.1.2. Validation samples

###### 2.1.1.2.1. Validation sample 1

The validation sample 1 included 2,310 participants from two French universities (University of Reims Champagne-Ardenne and University of Picardy Jules Verne), primarily of Caucasian, who responded to the questionnaire. Recruitment of university students was performed via an e-mail advertisement. All participants freely gave their formal, informed consent at the beginning of the study by using the consent button after reading the consent form. Criteria of inclusion were age between 18 and 25 and drinking 5 or more drinks (50 g) per week. No compensation was given.

###### 2.1.1.2.2. Validation sample 2

The validation sample 2 was selected based on BD indices for a brain imaging study with a total of 120 students (60 males and 60 females). All participants freely signed the inform consent form at the beginning of the study. This sample of 120 came from a broader sample of 391 participants (age, 18 to 24 years; 215 females and 176 males, all Caucasian regarding the genetic part of the study) among students at two French universities (Amiens and Reims). Inclusion and exclusion criteria fulfilled the requirements of the behavioral, brain imaging and genetic parts of the study.

### 2.2. Procedures and measurements

The online survey used for all three samples assessed demographic characteristics (age, gender, academic level and discipline, and living situation), drug use (alcohol, cigarettes, and cannabis), AUDIT score (Gache et al., 2005) and Binge drinking score (Mehrabian and Russell, 1978) (Tables 1, 2).

**Table 1:** Demographic data and drug consumption for the learning sample.

		*N*	%
Gender	M	290	22.7
	F	987	77.3
	Total	1,277	
	Mean ± sem		
Age	21.13 ± 0.05		
<21		530	41.5
21–23		571	44.7
≥24		176	13.8
Academic year			
First-year		277	21.7
Second-year		290	22.7
Third-year		268	21.0
Fourth-year		248	19.4
Fifth-year		194	15.2
Age at first alcohol consumption	15.05 ± 0.05		
Cigarette			
Smokers		331	25.9
Non-smokers		946	74.1

**Table 2:** Comparison of the learning sample with the validation sample 1 and the validation sample 2.

		Learning sample		Validation sample 1		Learning sample vs. Validation sample 1	Validation sample 2		Learning sample vs. Validation sample 2
		*N*	%	*N*	%	*p*	*N*	%	*p*
Gender	Distribution					<0.0001***			<0.0001***
	M	290	22.7	952	41.2		60	41.2	
	F	987	77.3	1,358	58.8		60	58.8	
Age	Mean ± sem Min-Max	21.13 ± 0.05 18–26		20.29 ± 0.05 17–30		<0.0001***	21.28 ± 0.16 18–25		<0.05*
	Distribution					<0.0001***			<0.05*
	< 21	530	41.47	1,444	62.5		44	36.7	
	21–23	571	44.70	663	28.7		59	49.2	
	≥ 24	176	13.8	203	8.8		17	14.1	
AUDIT	Mean ± sem Min-Max	7.12 ± 0.14 1–29		7.06 ± 0.113 0–33		= 0.174	9.19 ± 0.58 0–30		<0.0001***
	Distribution of the different levels					<0.0001***			<0.0001***
	Low risk	570	44.6	1,370	59.9		56	46.7	
	Hazardous	541	42.4	622	26.9		33	27.5	
	High risk	166	13.1	318	13.8		30	25	
BD score	Mean ± sem Min-Max	13.58 ± 0.34 1.32–106		20.07 ± 0.40 1.33–172		<0.0001***	28.26 ± 2.35 0–132		<0.0001***
	Distribution of the different groups of drinking					<0.0001***			<0.0001***
	Social	913	71.5	1,293	56		63	52.5	
	Intermediate	252	19.7	413	17.9		3	2.5	
	Binge	112	8.8	604	26.1		54	45.0	

AUDIT, Alcohol Use Disorder Identification Test. BD Score, Binge drinking score. Comparison between means were performed using the Student t test. Comparison between distribution were conducted using the Chi square. AUDIT groups were typically defined as follows: low risk level of dependance score < 7 for men and < 6 for women, hazardous level of dependance score ≥ 7 and for men and ≥ 6 for women and < 13, high risk level of dependance score ≥ 13. Binge drinking score is defined as < 15 for social drinking group, ≥17 and < 23 for intermediate drinking group and ≥ 13 for binge drinking group with Binge score = (4 × AUQ 10) + AUQ 11 + (0.2 × AUQ 12) with AUQ 10: When you drink, how fast do you drink?; AUQ 11: How many times have you been drunk in the last 6 months? and AUQ 12: What percentage of the times that you drink do you get drunk? (Townshend and Duka, 2005).

### 2.3. Alcohol-related measures

#### 2.3.1. Alcohol use disorders identification test

The AUDIT is a 10-item screening tool developed by the WHO to assess hazardous alcohol consumption, drinking behaviors, and alcohol-related problems (Saunders et al., 1993; Gache et al., 2005). AUDIT is a self-assessment questionnaire that measures frequency and quantity of alcohol consumed (such as the frequency of consuming 6+ drinks), behaviors associated with alcohol use, and negative outcomes related to alcohol consumption. *When you drink, how fast do you drink*? Total AUDIT scores were calculated by adding the scores for all 10 items (Saunders et al., 1993). The Cronbach’s alpha for the full sample on this scale was 0.792.

#### 2.3.2. The alcohol use questionnaire-revised (AUQ-R)

We used a French version of the revised version of the Alcohol Use Questionnaire (Townshend and Duka, 2002) initially developed by Mehrabian and Russell (1978). This version allows for the calculation of weekly level of alcohol use (units of alcohol by week, considering that in France 1 unit of alcohol is defined as 10 g of ethanol) and a binge score. This score was calculated for all participants on the basis of the information provided regarding: speed of drinking (average drinks per hour), number of times being drunk in the previous 6 month, and percentage of times getting drunk when drinking (for more details, see Townshend and Duka, 2002; Gierski et al., 2015).

#### 2.3.3. Alcohol purchase task (for behavioral economic indices)

The Alcohol purchase task (APT) (Murphy and MacKillop, 2006) is a self-report measure that assesses behavioral economic demand for alcohol, or consumption as a function of price. Demand indices on the APT are strongly correlated with clinical alcohol use (Zvorsky et al., 2019; Martínez-Loredo et al., 2021). The APT asks participants to read a vignette describing a typical alcohol-drinking context and report how many standard drinks they would consume at a variety of prices. In the current study, the vignette specified a 5-h drinking occasion and the prices ranged from 0 to 20 euros. The consumption data were screened for violations of trend, bounce, and reversals from zero using the criteria of Stein et al. (2015). Among the 1,261 participants who had APT data, 82 were excluded for one or more violation, leaving a final sample of 1,179. Behavioral economic parameters included the observed indices of intensity (reported consumption at zero price), Omax (maximum product of price × consumption) Pmax (price at Omax), and breakpoint-1 (BP1) (the highest price with non-zero consumption). These parameters were calculated for each participant using the Foster and Reed Excel tool (Foster et al., 2020). Finally, participant-level derived behavioral economic parameters of Q0 (derived intensity) and alpha (rate of change in elasticity) were produced using the exponentiated demand function of Koffarnus et al. (2015) (6), Q = Q0 * 10^(k(ê(−alpha * Q0 * C) – 1)), with the zero euro price replaced with 0.01, and the span parameter (k) set to 3.20, which represented the highest range of participant-level consumption in log units plus 0.50. For each participant, these alpha and k values were used to produce Essential Value (EV), a standardized measure of reinforcing value, with EV = 1/(100 * alpha * k1.5) (Hursh and Roma, 2013).

### 2.4. Selection of items for clustering

Cluster items were selected from the AUDIT and AUQ-R to capture a variety of BD-associated characteristics (1) consumption frequency (*How often do you have a drink containing alcohol*?), (2) drinks per typical day (*How many drinks containing alcohol do you have on a typical day when you are drinking*?), (3) frequency of consuming 6+ drinks (*How often do you have six or more drinks on one occasion*?), (4) consumption speed (*When you drink, how fast do you drink*?), (5) drunkenness frequency (*How many times have you been drunk in the last 6 months?*), (6) proportion of drunkenness episodes out of 10 drinking occasions (*What percentage of the times that you drink do you get drunk*? reported on a scale of 10 occasions), (7) proportion of hangover episodes out of 10 drinking occasions (*What percentage of the times you drink have you had a hangover*? reported on a scale of 10 occasions).

### 2.5. Statistical analyses

For descriptive statistics, quantitative variables were expressed as mean ± standard error of the mean (SEM) and qualitative variables were presented as percentages. Comparisons between samples were performed using *χ*^2^ tests for qualitative variables and ANOVA or Student’s test for quantitative variables. Comparison and analyze were conducted with Student *t*-test, ANOVA and chi-square performed with SPSS, version 23.

The particularity of our analysis is that the dependent variable (Binge group) is not yet available at the beginning of the analyses. Therefore, we first used Kmeans to classify patients and then used the partial proportional odds to internally reproduce the Kmeans results before testing the generalizability of our results in independent samples without re-running the Kmeans algorithm. The external validation step’s aim was to evaluate the robustness and consistency of our results found in the learning sample. The increasing mean values of the useful variables (variables included in the Kmeans) were compared between the four groups.

#### 2.5.1. *K*-means clustering method

**Figure 1:**
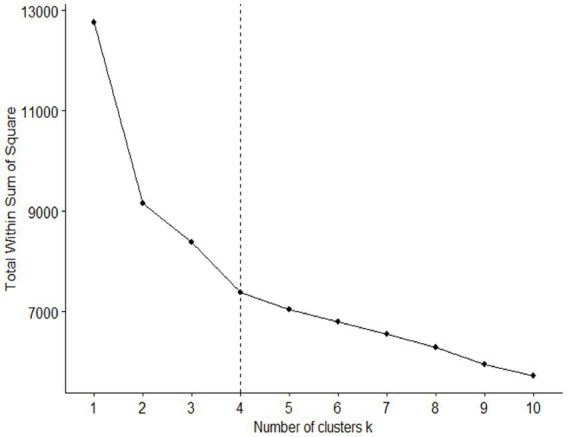
Optimal number of clusters. The Elbow method determined that four is the optimal number of clusters.

For exploratory determination of alcohol consumption patterns, the unsupervised *K*-means clustering method in the learning sample aimed to determine clusters of individuals who are as similar as possible (in contrast to individuals from different clusters, who should be as different as possible). Briefly, the *K*-means algorithm is an iterative algorithm that partitions the dataset into *K* pre-defined, distinct, and non-overlapping clusters whereby each individual belongs to only one cluster. The algorithm assigns data points to a cluster such that the sum of the squared distance between the individuals and the cluster’s centroid (i.e., the arithmetic means of all the individuals that belong to that cluster) is at its minimum. The less variation within clusters, the more homogeneous the individuals are within the same cluster. The optimal number of clusters was evaluated using the Elbow method (Figure 1). Afterward each participant had been classified in his/her corresponding cluster, we calculated cluster means for each of the variables used in the *K*-means method. Then, we ranked the clusters according to the progression of variables (Table 3).

**Table 3:** Cluster means for each of the variables used in the *K*-means method for the four groups in the learning sample.

	Consumption frequency	Number of drinks per typical day	6-drink frequency	Consumption speed	Drunkenness frequency	Number of drunkenness episodes/10 drinking occasions	Number of hangovers/10 drinking occasions
1 (*n* = 721)	1.79	0.70	0.90	1.47	0.93	1.24	1.23
2 (*n* = 404)	2.19	1.54	1.89	2.32	5.82	4.95	4.15
3 (*n* = 106)	2.60	2.04	2.66	2.85	19.28	6.10	4.65
4 (*n* = 46)	3.02	2.76	2.65	3.17	39.26	6.52	3.94

Cluster variables were selected from the AUDIT and AUQ-R questionnaires: (1) Consumption frequency or AUDIT 1 “How often do you have a drink containning alcohol?”, (2) Drinks per typical day or AUDIT 2 “How manny drinks containing alcohol do you have on a typical day when you are drinking?”, (3) 6 drinks frequency or AUDIT 3 “How often do you have six or more drinks on one occasion?”, (4) Consumption speed or AUQ 10 “When you drink, how fast do you drink?”, (5) Drunkenness frequency or AUQ 11 “How many times have you been drunk inn the last 6 months?”, (6) Number of drunkenness episodes/10 drinnkinng occasions or AUQ 12 “What percentage of the times that you drink do you get drunk?” reported on a scale of 10 occasions, (7) Number of hangovers/10 drinking occasions or “What percentage of the times you drink have you had a hangover?” reported on a scale of 10 occasions.

#### 2.5.2. Partial proportional odds model

We first modeled the probability of group membership using a PPOM allowing for unequal regression coefficients with the *K*-means clusters variable as the dependent variables. PPOM is a regression modeling technique aiming to assess the association between independent predictors and an ordinal outcome variable. After model building, the predictors’ regression coefficients could be used to predict the outcome. Instead, proportional odds model (POM), PPOM assumes that the effect some independent variable may not be uniform for all levels of the dependent variable (group variable). After determining the regression equations using variables significant at the 0.05 level of alpha on the PPOM, we estimated the probability *P_i_ (Group ≥ j)* for individual *i* to belong to at least group *j*. Thus for the probability of individual *i* to belong to group *j* = 2, …, *K-1* was estimated by *P_i_ (Group ≥ j)* – *P_i_ (Group ≥ j + 1)*, except for the first and last groups (Group 1 and Group 4) for which the probability of membership was estimated as 1 – Pi (Group ≥2) and Pi (Group ≥4) respectively. Then, two phases of validation were followed (Moons et al., 2012a,b). For internal validation in this sample, each individual was assigned to the group for which his/her probability of membership was the highest. Next, we compared the group membership from the clustering method and the group membership estimated from the PPOM model with a calculation of misclassification error from the PPOM model using the groups derived from clustering as the gold standard. For external validation, individuals in the two validation samples were assigned to the group for which their probability of belonging estimated from the PPOM model was the highest. Next, for the validation database we estimated the means for each of the PPOM independent variables across the groups.

PPOM and model validation were performed with R software, version 3.4.0 (R Foundation for Statistical Computing, Vienna, Austria) through the RStudio interface, version 1.0.143. The *K*-means and function were used in the learning database and the VGAM library for PPOM modeling.

### 2.6. Ethical approval

The personal identity of the participants completing the anonymous questionnaire was unknown to the researcher. Each participant provided electronic informed consent after reading the consent form, which described the purpose of research, participation, procedure, confidentiality, and researcher’s contact information. Raw data were stored on a computer not connected to an internet network. We removed the access link to the data collected online at the end of the study. Learning sample was part of a study approved by the regional ethics committee (Comité de Protection des Personnes Nord-Ouest II). Validation samples were part of a bigger study approved by the regional ethics committee (Comité de Protection des Personnes Est I).

## 3. Results

### 3.1. Demographic data and description of drugs consumptions for the learning sample

We calculated the mean and SEM of each continuous variable (e.g., age, age at first alcohol use) and the percentages on each demographic item – age, gender, age of first consumption, year in school (Table 2).

### 3.2. Comparison of the learning population with the two validation samples

The 2 validation samples differ significantly from the learning sample with respect to parameters such as the gender ratio and consumption parameters (AUDIT and BD group distribution) (Table 2). These significant differences between the 3 samples reinforce the validity of our model.

### 3.3. Cluster analyses

Using the Elbow method, we determined four clusters, designated groups 1, 2, 3, and 4 (Figure 1). This number of groups resulted from maximizing the number of individuals in each group.

### 3.4. Partial proportional odds model

#### 3.4.1. Major factors in the classification

On the 7 items initially selected (see *Selection of items for clustering section*), we found that 5 of them (1) 6 drinks frequency or AUDIT 3 “How often do you have six or more drinks on one occasion?”, (2) Consumption speed or AUQ 10 “*When you drink, how fast do you drink?*”, (3) Drunkenness frequency or AUQ 11 “*How many times have you been drunk inn the last 6 months?*”, (4) Number of drunkenness episodes/10 drinnkinng occasions or AUQ 12 “*What percentage of the times that you drink do you get drunk?*” reported on a scale of 10 occasions, (5) Number of hangovers/10 drinking occasions or “*What percentage of the times you drink have you had a hangover?*” reported on a scale of 10 occasions, were significant at the 0.05 level on the PPOM analyses (Table 4). For the final selection of items, we considered multicollinearity, that is, strongly correlated dependent variables. In particular, of the first 3 AUDIT items, we selected item 3 (6-drink frequency), which had the most impact on our model (Table 4). Mean scores on each of the five significant items (Table 3) were calculated for each group. The results show that the mean scores increased gradually from group 1 to group 4 (Table 3), with minor exceptions from group 3 to 4.

**Table 4:** Results of the partial proportional odds model (PPOM) for the variable of group membership (the four groups derived from the *K*-means model) as the dependent variable.

PPOM: significant items					
	Group	Regression coefficients	Std. Error	*Z* value	*p* value
AUDIT 1 - Consumption frequency	≥2, ≥ 3, = 4	0.18809	0.15811	1.190	0.23418
AUDIT 2 - Drinks per typical day	≥2, ≥ 3, = 4	−0.01347	0.09327	−0.144	0.88520
AUDIT 3–6 drinks frequency	≥2, ≥ 3, = 4	0.42646	0.14719	2.897	<0.01**
AUQ 10 - Consumption speed	≥2	0.20057	0.10606	1.891	0.05859
	≥ 3	0.07370	0.12862	0.573	0.56665
	= 4	−0.12883	0.20744	−0.621	0.53455
AUQ 11 - Drunkenness frequency	≥2, ≥ 3, = 4	0.33708	0.02394	14.083	<0.001***
AUQ 12 – Number of drunkenness episodes (out of 10 drinking occasions)	≥2	0.68408	0.06473	10.568	<0.001***
	≥ 3	0.11046	0.08512	1.298	0.19439
	= 4	0.02215	0.13689	0.162	0.87146
Number of hangovers (out of 10 drinking occasions)	≥2	0.36077	0.05103	7.070	<0.001***
	≥ 3	0.05129	0.06900	0.743	0.45725
	≥ 4	−0.34092	0.12573	−2.712	<0.01**

**p < 0.01; ***p < 0.001. The items “6 drinks frequency,” “Drunkenness frequency,” “Number of drunkenness episodes (out of 10 drinking occasions)” and “Number of hangovers (out of 10 drinking occasions)” were significant at a 5% level of significance, and the item “Consumption speed” tended to a 5% level of significance. This result indicated that they were the most important deterministic factors in the classification. The three independent variables “Consumption speed,” “Number of drunkenness (out of 10)” and “Number of hangover (out of 10)” did not meet the proportional odds assumption and had different coefficients for each of three modeled probabilities, P (Group ≥ 2), P (Group ≥ 3) and P (Group = 4).

#### 3.4.2. Cumulative probability equations

The partial proportional model generated the four equations below. These equations permit calculation of the probability of belonging to each group for each individual.


$$\begin{array}{c} P\left(Group\geq 2\right)=\frac{e^{-5.87+0.43*{\mathrm{Freq}}_{\mathrm{six}-\mathrm{drinks}}+0.20*\mathrm{speed}+0.34*{\mathrm{Freq}}_{\mathrm{drunkeness}}+0.68*{\mathrm{Number}}_{\mathrm{Drunkeness}\;\mathrm{on}\;10}+0.36*{\mathrm{Number}}_{\mathrm{Hangover}\;\mathrm{on}\;10}}}{\left(1+e^{-5.87+0.43*{\mathrm{Freq}}_{\mathrm{six}-\mathrm{drinks}}+0.20*\mathrm{speed}+0.34*{\mathrm{Freq}}_{\mathrm{drunkeness}}+0.68*{\mathrm{Number}}_{\mathrm{Drunkeness}\;\mathrm{on}\;10}+0.36*{\mathrm{Number}}_{\mathrm{Hangover}\;\mathrm{on}\;10}}\right)}\\ P\left(Group\geq 3\right)=\frac{e^{-7.37+0.43*{\mathrm{Freq}}_{\mathrm{six}-\mathrm{drinks}}+0.07*\mathrm{speed}+0.34*{\mathrm{Freq}}_{\mathrm{drunkeness}}+0.11*{\mathrm{Number}}_{\mathrm{Drunkeness}\;\mathrm{on}\;10}+0.05*{\mathrm{Number}}_{\mathrm{Hangover}\;\mathrm{on}\;10}}}{\left(1+e^{-7.37+0.43*{\mathrm{Freq}}_{\mathrm{six}-\mathrm{drinks}}+0.07*\mathrm{speed}+0.34*{\mathrm{Freq}}_{\mathrm{drunkeness}}+0.11*{\mathrm{Number}}_{\mathrm{Drunkeness}\;\mathrm{on}\;10}+0.05*{\mathrm{Number}}_{\mathrm{Hangover}\;\mathrm{on}\;10}}\right)}\\ P\left(Group=4\right)=\frac{e^{-8.46+0.43*{\mathrm{Freq}}_{\mathrm{six}-\mathrm{drinks}}-0.13*\mathrm{speed}+0.34*{\mathrm{Freq}}_{\mathrm{drunkeness}}+0.02*{\mathrm{Number}}_{\mathrm{Drunkeness}\;\mathrm{on}\;10}-0.34*{\mathrm{Number}}_{\mathrm{Hangover}\;\mathrm{on}\;10}}}{\left(1+e^{-8,46+0.43*{\mathrm{Freq}}_{\mathrm{six}-\mathrm{drinks}}-0.13*\mathrm{speed}+0.34*{\mathrm{Freq}}_{\mathrm{drunkeness}}+0.02*{\mathrm{Number}}_{\mathrm{Drunkeness}\;\mathrm{on}\;10}-0.34*{\mathrm{Percent}}_{\mathrm{Hangover}\;\mathrm{on}\;10}}\right)}\\ P\left(Group=j\right)=P\left(Group\geq j\right)–P\left(Group\geq j+1\right)\;for\;j=2\;or\;3,\\ P\left(Group=1\right)=1-P\left(Group\geq 2\right)\;and\;P\left(Group=4\right)=P\left(Group\geq 4\right) \end{array}$$

#### 3.4.3. Internal validation

The risk of misclassification of one individual in each group was obtained by cross-referencing the result from the *K*-means method with the resultant prediction of the equations above reapplied to the learning sample. This level of risk of misclassification indicates a good internal validation of our model. The internal validation with low risk of misclassification of the PPOM confirms the reliability of the distribution (Table 5).

**Table 5:** Concordance between the results from the *K*-means and the PPOM model for the learning sample.

PPOM grouping					
	*N*	Group 1	Group 2	Group 3	Group 4
*K*-means grouping	Group 1	705	16	0	0
	Group 2	33	370	1	0
	Group 3	0	11	90	5
	Group 4	0	0	0	46

*N*, number of participants. Misclassification risks were: 16/721 = 2.2% in group 1; 34/404 = 8.4% in group 2; 16/106 = 15.1% in group 3; 0 in group 4.

### 3.5. External validation

We tested the reproducibility of our classification method by comparing the mean values of the 5 identified items in the learning sample with their mean values on these items obtained in each group for the validation samples (i.e., samples 2 and 3 in the present study).

The probability of belonging to each group was calculated for each individual in the validation samples. The means for each item were calculated for the two validation samples tested. Despite the marked disparities between the 3 samples we tested, the means obtained for each item in each group were very similar between the 3 samples, indicating the strong validity of our model (compare Table 6 with Table 3).

**Table 6:** Cluster means on each of the variables for validation samples 1 and 2.

Groups	6 drinks frequency	Consumption speed	Drunkenness frequency	Number of drunkenness (out of 10)	Number of hangovers (out of 10)
Validation sample 1					
1 (*n* = 1,496)	0.86	1.72	1.05	1.36	1.50
2 (*n* = 559)	1.94	2.94	6.27	5.09	4.28
3 (*n* = 125)	2.70	3.60	19.18	6.48	4.97
4 (*n* = 121)	2.94	4.00	43.08	6.88	4.45
Validation sample 2					
1 (*n* = 53)	1.13	1.51	1.66	1.11	1.37
2 (*n* = 25)	2.00	2.96	8.60	4.84	3.86
3 (*n* = 10)	2.40	3.10	19.20	5.70	4.70
4 (*n* = 23)	3.02	3.48	43.17	6.87	4.44

By applying the equations in our 2 validation samples, mean results were identical to those of the learning sample (Table 3). Significant items means gradually increased from group 1 to group 4 (except for the number of hangovers). This result illustrates the reproducibility of the equations.

### 3.6. The influence of gender

**Figure 2:**
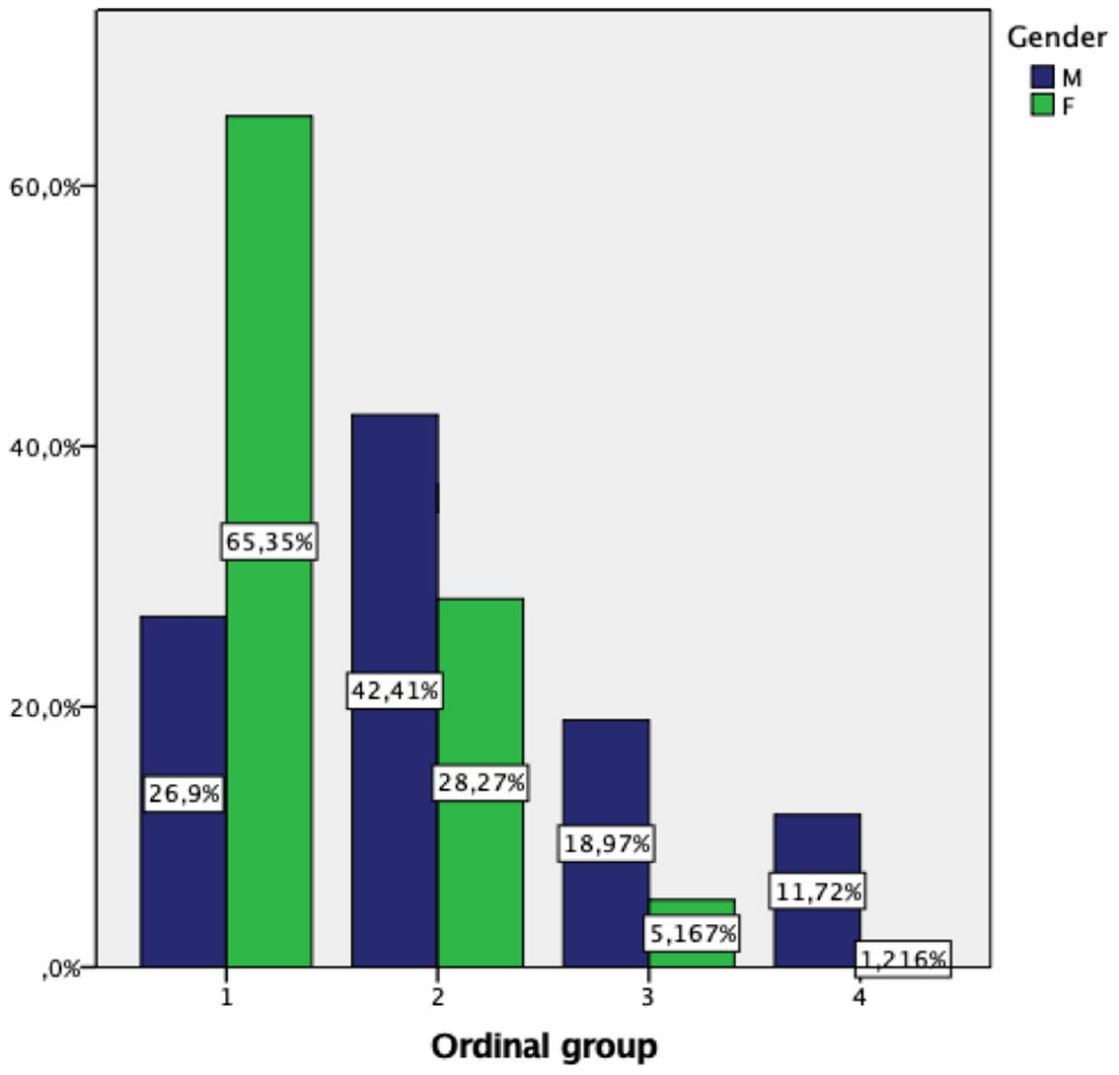
The percentage of women was highest in group 1 (65.35%) and decreased sharply in groups 2 (28.27%), 3 (5.17%) and 4 (1.22%). The percentage of men was highest in group 2 (42.41%) and comparatively higher than the percentage of women in groups 3 (18.97%) and 4 (1.22%).

We next checked whether gender affected the specificity of the group classification in the learning sample (see Figure 2 for gender distribution) by calculating the risk of misclassification with or without the inclusion of gender in the PPOM model (Tables 7A,B). Overall, we found that the integration of the gender parameter in the PPOM did not improve the specificity of the classification. More precisely, the inclusion of the gender parameter into the equation had no effect on the classification in group 1 (2.1% versus 2.2%) and group 4 (0 versus 0). However, our analysis revealed a slight difference for groups 2 (8.4% versus 9.2%) and group 3 (15.1% versus 16%) (Table 6). Therefore, our model of classification is valid regardless of gender.

**Table 7:** A and B: Influence of the gender parameter.

(A) Risk of misclassification without the gender parameter						
	Derivation					
	*N*	Group 1	Group 2	Group 3	Group 4	Error
*K*-means	Group 1	705	16	0	0	2.2%
	Group 2	33	370	1	0	8.4%
	Group 3	0	11	90	5	15.1%
	Group 4	0	0	0	46	0

(B) Risk of misclassification with the gender parameter						
	Derivation					
	N^a^	Group 1	Group 2	Group 3	Group 4	Error
K-means	Group 1	706	15	0	0	2.1%
	Group 2	34	370	0	0	9.2%
	Group 3	0	10	89	7	16%
	Group 4	0	0	0	46	0

*N*, number of participants. Error risk did not differ significantly with the inclusion of gender.

### 3.7. Group characterization

#### 3.7.1. Comparison between the 4 groups

We next analyzed the 4 groups with known items from the AUDIT questionnaire (Table 8) and behavioral economic indices from the APT (Table 9) in order to evaluate the concordance of our groups with other indices of alcohol use, severity, and demand. In general, the severity of the alcohol consumption or its consequences increases from group 1 to 4. Moreover, there was no significant difference by gender, particularly for groups 3 and 4.

**Table 8:** Alcohol consumption and alcohol-related behaviors data by AUDIT items distributed among the four groups.

Group		1	2	3	4	*p*	I.R.R. 4 vs. 3
		%	%	%	%		
Gender						<0.0001***	
	M	10.8	30.6	51.9	73.9		1.4
	F	89.2	69.4	48.1	26.1		0.5
First drink before 12 years old		5.1	6.2	8.5	17.4	<0.0001***	
Binge Group according to Binge Score							
	Mean ± sem	7.07 ± 4.02	16.14 ± 5.65	31.90 ± 6.85	53.26 ± 15.19	<0.0001***	
						<0.0001***	
	Social drinkers	96.4	53.7	0	0		0
	Intermediate drinkers	3.6	43.5	48.1	0		0
	Binge drinkers	0	2.7	51.9	100		1.9
Audit Group							
	Mean ± sem	4.53 ± 0.41	9.03 ± 3.80	13.47 ± 4.8	16.57 ± 5.4	<0.0001***	
						<0.0001***	
	Low risk level	69.7	15.4	1.9	2.2		1.1
	Hazardous level	29.5	65.7	50	23.9		0.5
	High risk level	0.8	18.9	48.1	73.9		1.5
AUDIT 2 *Number of drinks containing alcohol on a typical day*						<0.0001***	
	1 or 2	48.5	17.7	12.3	10.9		0.9
	3 or 4	35.4	28.4	17	10.9		0.6
	5 or 6	14.8	45.3	48.1	23.9		0.5
	7. 8. or 9	0	0	0	0		0
	10 or more	1.2	8.7	22.6	54.3		2.4
AUDIT 4 *Not able to stop drinking once you had started*						<0.0001***	
	Never	87.3	60.7	41.5	41.3		1.0
	Less than monthly	11.8	26.4	24.5	17.4		0.7
	Monthly	0.6	11.4	17	26.1		1.5
	Weekly	0.1	1.5	16	15.2		1.0
	Daily or almost daily	0.3	0	0.9	0		0
AUDIT 5 *Failed to do what is normally expected*						<0.0001***	
	Never	80.6	51.5	26.4	21.7		0.8
	Less than monthly	18.9	39.3	44.3	39.1		0.9
	Monthly	0.4	8	20.8	23.9		1.1
	Weekly	0	1.2	8.5	15.2		1.8
AUDIT 6 *Needed a first drink in the morning*						<0.0001***	
	Never	97.1	85.1	66	47.8		0.7
	Less than monthly	2.4	10.4	17.9	26.1		1.5
	Monthly	0.4	4.5	13.2	15.2		1.1
	Weekly	0.1	0	1.9	10.9		5.7
	Daily or almost daily	0	0	0.9	0		0
AUDIT 7 *Feeling of guilt or remorse after drinking*						<0.0001***	
	Never	72.3	42.8	34.9	43.5		1.2
	Less than monthly	26.3	46.3	48.1	37		0.8
	Monthly	1	9.7	14.2	17.4		1.2
	Weekly	0.4	1	1.9	0		0
	Daily or almost daily	0	0.2	0.9	2.2		2.4
AUDIT 8 *Unable to remember what happened the night before*						<0.0001***	
	Never	82.6	51	25.5	13		0.5
	Less than monthly	16.6	40.5	42.5	45.7		1.1
	Monthly	0.3	7.5	27.4	34.8		1.3
	Weekly	0	0.2	3.8	6.5		1.7
	Daily or almost daily	0.6	0.7	0.9	0		0
AUDIT 9 *Someone else been injured*						<0.0001***	
	No	92.4	82.8	71.7	63		0.9
	Yes but not in the last year	5.5	9	17.9	10.9		0.6
	Yes during the last year	2.1	8.2	10.4	26.1		2.5
AUDIT 10 *Relative/friend/ doctor concerned about your drinking*						<0.0001***	
	No	97.1	89.8	80.2	60.9		0.8
	Yes, but not in the last year	1.7	5.2	6.6	10.9		1.7
	Yes, during the last year	1.2	5	13.2	28.3		2.2
AUQ 11 *Number of times being drunk in the last 6 months?*						<0.0001***	
	1 or less	47.4	0.7	0	0		
	1 to 5	52.6	49.5	0	0		
	6 to 25	0	49.8	100	0		
	30 to 50	0	0	0	100		
Consumption of 4 or more drinks per hour	2.7	12	19.8	32.8			1.7

IRR, incidence rate ratio. Comparison between means were performed using ANOVA. Comparison between distribution were conducted using the Chi square. The 4 groups are depicted by the increasing severity of the alcohol-related consumption and consequences items. IRR illustrates differences between group 3 and group 4. ***p < 0.0001.

**Table 9:** Comparison of the behavioral economic indices among the 4 groups.

Group	1	2	3	4	*p*
Breakpoint-0	11.31 ± 0.18	11.77 ± 0.25	12.40 ± 0.45	10.89 ± 0.68	0.089
Breakpoint-1	11.15 ± 0.18	12.07 ± 0.25	12.92 ± 0.4	11.84 ± 0.79	<0.001**
Observed intensity	6.13 ± 0.17	10.22 ± 0.37	12.31 ± 0.62	16.13 ± 1.42	<0.0001***
Observed Omax	15.97 ± 0.38	20.92 ± 0.75	25.86 ± 2.01	27.00 ± 2.28	<0.0001***
Observed Pmax	6.61 ± 0.16	6.07 ± 0.20	6.48 ± 0.47	6.02 ± 0.63	0.212
*Q*_0_	6.36 ± 0.16	10.45 ± 0.36	12.37 ± 0.58	16.66 ± 1.40	<0.0001***
Alpha	0.014 ± 0.007	0.005 ± 0.0001	0.004 ± 0.0003	0.005 ± 0.0013	0.693

Values are presented as Mean ± SEM. Comparison between means were performed using ANOVA. Q_0_ and alpha represent the derived indices of demand intensity and rate of change in elasticity, respectively, produced from fits of the Koffarnus et al. (2015) exponentiated demand equation to the participant-level demand data Observed intensity represents reported consumption at zero price; breakpoint-0 is the first price at which consumption is suppressed; breakpoint-1 is the last price of non-zero consumption; observed Omax is the maximum product of price × consumption; and observed Pmax is the price at which observed Omax occurs. Group 4 displayed significantly higher demand intensity (both observed and derived) compared with group 3 suggesting a more problematic use of alcohol in group 4, as seen in other population with AUD. Since we did not observe difference in elasticity (alpha) between groups, our results do not support an addictive behavior in the binge drinking group. **p < 0.001; ***p < 0.0001.

With respect to the alcohol demand indices, we observed a significant omnibus effect of group on breakpoint-1, observed intensity, observed *O*_max_, and *Q*_0_ (derived intensity), with increases in each of these demand indices across groups 1 to 4, except for breakpoint-1 (Table 9).

#### 3.7.2. Comparison of groups 3 and 4

All of the alcohol consumption criteria were significantly different between drinking groups 3 and 4 (Table 8). As shown in Table 8, comparing the incidence risk ratios (IRRs) for groups 3 and 4 reveals two different drinking profiles, which also are reflected in the behavioral economic data (Tables 9, 10). Specifically, the comparison of alcohol demand indices between groups 3 and 4 revealed significant differences in both intensity measures (observed and derived *Q*_0_), with significantly higher demand intensity for group 4.

**Table 10:** Comparison of consumption parameters and demand indices between Groups 3 and 4.

Group	3	4	*p*
	Mean ± sem	Mean ± sem	
Consumption criteria			
Units per week	9.68 ± 0.73	14.26 ± 1.30	<0.0001****
Pints of beer per week	5.62 ± 0.507	9.65 ± 1.043	<0.0001****
Spirits per week	4.09 ± 0.388	6.11 ± 0.865	<0.05*
AUDIT Score	13.47 ± 0.461	16.57 ± 0.800	<0.0001***
AUDIT 6 - first drink in the morning	0.54 ± 0.084	0.89 ± 0.153	<0.05*
Binge score	32 ± 0.67	53 ± 2.24	<0.0001****
Demand parameters			
Breakpoint 0	12.40 ± 0.45	10.89 ± 0.68	0.063
Breakpoint 1	12.92 ± 0.4	11.84 ± 0.79	0.234
Observed intensity	12.31 ± 0.62	16.13 ± 1.42	<0.005**
Observed Omax	25.86 ± 2.01	27.00 ± 2.28	0.735
Observed Pmax	6.48 ± 0.47	6.02 ± 0.63	0.574
Q0	12.37 ± 0.58	16.66 ± 1.40	<0.00001***
Alpha	0.004 ± 0.0003	0.005 ± 0.0013	0.398

Values are presented as Mean ± SEM. Comparison between means were performed using Student *t*-test. *p < 0.05; **p < 0.005; ***p < 0.001; ****p < 0.0001.

#### 3.7.3. Comparison between genders in the four groups

Means of consumptions items (AUDIT Score, Binge drinking Score) and behavioral related to consumption items (Age of start of consumption, AUDIT 2, 4, 5, 6, 8, 9, 10 listed in Table 11) differ between men and women in groups 1 and 2. No significant differences were found in the same criteria between women and men in groups 3 and 4.

**Table 11:** Consumptions and behavioral measures related to consumption per group per sex.

	Group 1 *N* = 723 (56.62%)			Group 2 *N* = 402 (31.48%)			Group 3 *N* = 106 (8.30%)			Group 4 *N* = 46 (3.60%)		
Gender	M	F		M	F		M	F		M	F	
	*N* = 78 (26.90%)	*N* = 645 (65.34%)		*N* = 123 (42.41%)	*N* = 279 (28.27%)		*N* = 55 (18.96%)	*N* = 51 (5.16%)		*N* = 34 (11.7%)	*N* = 12 (1.22%)	
	Mean ± sem		*p*	Mean ± sem		*p*	Mean ± sem		*p*	Mean ± sem		*p*
Age of start of consumption	14.97 ± 0.252	15.45 ± 0.074	<0.05*	14.61 ± 0.168	14.90 ± 0.082	0.142	14.20 ± 0.179	14.12 ± 0.176	0.790	13.65 ± 0.286	14.58 ± 0.358	0.084
AUDIT	7.03 ± 0.385	4.23 ± 0.096	<0.0001***	11.17 ± 0.385	8.09 ± 0.189	<0.0001***	13.93 ± 0.66	12.98 ± 0.65	0.303	16.50 ± 0.926	16.75 ± 1.657	0.939
Binge Drinking Score	10.26 ± 0.487	6.68 ± 0.150	<0.0001***	17.83 ± 0.50	15.39 ± 0.33	<0.0001***	32.78 ± 0.91	30.95 ± 0.96	0.194	51.60 ± 2.06	57.98 ± 6.31	0.228
AUDIT 4 *Not able to stop drinking once you had started*	0.38 ± 0.084	0.11 ± 0.014	<0.0001***	1.10 ± 1.14	1.15 ± 1.13	<0.01*	1.22 ± 0.168	0.98 ± 0.144	0358	1.09 ± 0.186	1.33 ± 0.3767	0.585
AUDIT 5 *Failed to do what is normally expected*	0.27 ± 0.054	0.19 ± 0.016	0.095	0.73 ± 0.072	0.53 ± 0.038	<0.01*	1.05 ± 0.123	1.18 ± 0.124	0.469	1.29 ± 0.172	1.42 ± 0.288	0.740
AUDIT 6 *Needed a first drink in the morning*	0.13 ± 0.053	0.02 ± 0.007	<0.0001***	0.33 ± 0.057	0.13 ± 0.024	<0.0001***	0.45 ± 0.096	0.63 ± 0.140	0.230	0.91 ± 0.191	0.83 ± 0.241	0.766
AUDIT 8 *Unable to remember what happened the night before*	0.23 ± 0.048	0.19 ± 0.019	0.506	0.78 ± 0.068	0.51 ± 0.040	<0.001***	1.15 ± 0.123	1.10 ± 0.116	0.687	1.32 ± 0.138	1.42 ± 0.229	0.762
AUDIT 9 *Someone else been injured*	0.41 ± 0.117	0.17 ± 0.026	<0.01*	0.83 ± 0.128	0.37 ± 0.062	<0.0001***	0.87 ± 0.193	0.67 ± 0.174	0.400	1.29 ± 0.303	1.17 ± 0.520	0.862
AUDIT 10 *Relative or friend concerned about your drinking*	0.38 ± 0.127	0.05 ± 0.014	<0.0001***	0.62 ± 0.120	0.16 ± 0.041	<0.0001***	0.80 ± 0.198	0.51 ± 0.184	0.268	1.24 ± 0.293	1.67 ± 0.595	0.522
*Mediane*												
AUDIT 2 *How many drinks in a typical day*	1^£^	0^£^		2^£^	2^£^		3^£^	3^£^		3^£^	3^£^	

^£^0 = 1 or 2 drinks/typical day 3 = 7, 8 or 9 drinks/typical day. 1 = 3 or 4 drinks/typical day 4 = 10 or more drinks/typical day. 2 = 5 or 6 drinks/typical day. Means comparison were performed with ANOVA. *p < 0.05; **p < 0.005; ***p < 0.001; ****p < 0.0001.

## 4. Discussion

The goal of the current study was to develop an objective and simple tool for identification and characterization of BD for both genders based on a progressive severity. To our knowledge, this is the first study to propose an integrative model of BD that captures comprehensive features of BD and has led to a global definition combining 5-item (Appendix, online access to two tools: a population sample classification tool,^1^ and an individual characterisation tool^2^).

The analyses we performed on our student learning sample revealed 4 homogeneous groups, defined by 5 salient items that together, capture a set of key BD factors (Maurage et al., 2020) including quantitative aspects of consumption (quantity-frequency with “6-drink frequency”), behavior (speed of consumption), and physiology (frequency of drunkenness, frequency of hangover). As such, ours is the only existing model that combines all of these key factors in a single and consistent measure. Moreover, our use of the statistical PPOM validation has 2 key advantages: (i) it ensures an objective, reliable and reproducible model, and (ii) it avoids the duplication of highly correlated items and selects only salient and statistically significant factors (i.e., 6-drink frequency and not consumption frequency nor drinks per typical day). The internal validation and low classification error of the PPOM that we found confirm construct validity of the 4 groups (McAloney et al., 2013).

The magnitude of all 5 final items progressively increased from group 1 to group 4 (Table 3), with only minor exceptions between the two most severe groups (3 and 4). In addition, the regression equations effectively predicted a participant’s probability of belonging to each group. In contrast to the methods using cut-off delimitations in the identification of the population groups, the present approach does not constrain analysis of BD into a dichotomous variable (McAloney et al., 2013). Rather, our model describes a progressive severity to categorize the binge-drinking level of a given participant and accurately map the drinking pattern.

Following the PPOM validation, the external validation confirmed the reproducibility of our method and generalization to two additional samples (Debray et al., 2015; Ramspek et al., 2021). The external validation samples differed from the learning population in many ways, such as sample size, gender ratio, and alcohol consumption metrics (Table 2). Despite these differences, our findings reveal a strong reproducibility of our validation items and confirm that the model variables are robust and that the groups are consistent. Thus, unlike prior studies (Townshend and Duka, 2005), the current model provides a valid characterization of BD independent of the characteristics of the sample.

To further confirm the validity of the 4 groups, they were reassessed using known criteria related to alcohol use and consequences. Specifically, we found that percentage of participants in high consumption scores sub-groups (high BD and AUDIT scores) and high adverse consequences sub-groups (AUDIT 2, 4–10, 14; first drink before 12 years old; consumption of 4 or more drinks in one occasion) as well as means of consumption scores (Table 8), and behavioral economic indices (Table 9) gradually increased across groups. Our behavioral economic results confirm consumption criteria. Previous studies reported correlations between behavioral economic indices and risky drinking. APT indices are correlated with measure of alcohol use, alcohol-related consequences, and alcohol use disorder criteria (Gaume et al., 2022). In particular, the demand metrics of “intensity” (i.e., amount of alcohol consumed at zero or very low price, or “How much alcohol would you consume if alcohol were free?”) and Omax (i.e., maximum expenditure on alcohol in one occasion) predict clinical alcohol problems, beyond alcohol consumption alone (MacKillop and Murphy, 2007) and 6-month binge drinking and alcohol problems (Dennhardt et al., 2015). Furthermore, since we did not observe a difference in elasticity (alpha) between the groups, our results do not support addictive behavior in the binge drinking group.

These results highlight both the homogeneity within each group and the severity scale across groups. Therefore, we propose the following category labels based on the characteristics of each group: low-risk drinking (group 1), hazardous drinking (group 2), BD (group 3) and high-intensity BD (group 4). By differentiating binge drinking and high-intensity BD as we do for groups 3 and 4, respectively, our approach offers new opportunities to better identify and describe individuals with these drinking profiles (Gmel et al., 2007; Read et al., 2008; Patrick and Azar, 2018; Fairlie et al., 2019). Indeed, the present results reveal some distinctions. For example, compared to group 3, group 4 users are more likely to start drinking before the age of 12, consume more than 10 drinks (100 g) per occasion, fail to do what is expected, need a first drink in the morning, experience weekly black-outs, and drink 4 (40 g) or more drinks per hour. In addition, only group 4 users reported being drunk more than 30 times in the last 6 months meaning at least one episode of drunkenness per week (Table 8). Participants in group 4 also display greater behavioral economic demand, specifically with significantly higher demand intensity, consistently associated with clinical severity of alcohol use.

Results of consumptions items and behavioral economics items confirm the severity of the consumption-related consequences in the high-intensity BD. High-intensity BD is linked to alcohol-related injuries, alcohol poisoning, risky sexual behavior, vomiting, fainting, long-term damage (Patrick and Azar, 2018). The link between high-intensity BD and addictive behaviors will be further investigated. Because high-intensity BD is identified as a strong prospective marker of risk for AUD symptoms in adults (Patrick et al., 2021), our tool could be decisive in identifying this drinking profile early and thereby improving prevention (Enoch et al., 2006; Maurage et al., 2020). Considering the widespread use of BD in adolescents and young adults, the accurate identification of High-intensity BD is highly relevant for providing personalized feedback and awareness-raising to these populations.

Another strength of our model is its applicability across genders (Table 6). In fact, adding the gender factor to the regression equations had no effect on misclassification error, indicating stability of a participant’s classification in one of the 4 groups, regardless of gender. In our exploratory analyses of gender differences, we found differences in drinking behavior within groups 1 and 2, but not in the more severe groups (Table 11). Given that social factors impact drinking habits differently between genders (Graham et al., 1998; Wilsnack et al., 2018), these results may indicate that the impact of such factors is stronger in the less severe groups compared with the more severe ones. The reliability of our model for each gender opens up the ability to study gender-specific differences in consumption behavior with a high degree of confidence.

Our results reveal three additional aspects of interest. First, the frequency of hangovers differentiates low risk drinking from other profiles. Specifically, we found that groups 2, 3, and 4 reported significantly more frequent hangovers compared with group 1, suggesting that hangover is specific to risky drinking patterns; however, we also found that the frequency of hangover was significantly *lower* in group 4 compared with group 3. This finding indicates that hangover does not linearly increase with severity of consumption (Swift and Davidson, 1998) and that high-intensity BD group may not be motivated by experiencing hangover. Second, the frequency of drunkenness is weighted heavily and effectively differentiates hazardous drinking from BD and, even more powerfully, BD from high-intensity BD (Tables 3, 6). These findings confirm that drunkenness is a strong marker of BD severity and should be taken into account in such definitions, as noted by others (Lannoy et al., 2021). Third, and perhaps most importantly, our model confirms that BD should be considered over time and not as a single instance of behavior (i.e., not simply a single instance of 4+/5+ drinks). As noted by Gmel et al. (2011), the frequency of “risky single-occasion drinking” (RSOD) may differ widely between groups, with moderate drinkers displaying RSOD rarely (Gmel et al., 2011). A model that considers the chronicity of BD episodes is necessary to discriminate consequences of occasional BD from chronic BD, in terms of (a) severity of harm and tolerance and (b) experience in managing these effects at equivalent levels of blood alcohol concentrations (Gmel et al., 2011). Our model addresses both of these needs and fills an important gap noted by several others.

Despite its strengths, our model has some limitations. For example, the learning sample and both validation samples originated from only one country. Although the three samples differed in many ways (such as average level of consumption and gender distribution), replication is warranted in future studies, with external geographical validation, to confirm that the model remains valid despite cultural differences. Moreover, a larger sample would allow the recruitment of more participants displaying high-intensity BD to better characterize this type of drinking. Reliance on self-report is also a limitation (Creswell et al., 2020) since young adults may minimize or exaggerate their drinking levels. Another limitation of this study is that it is conducted in a student population. Future studies may look at other populations in terms of age and geographical region. Our model could indeed address the growing need to study BD in middle-aged adults, particularly among women (age 30–44), for whom the incidence of BD has nearly doubled in the past decade (Han et al., 2019; Patrick et al., 2019). Finally, the reference period for the AUDIT is 1 year, and thus the recall for past drinking experiences may have been less reliable on these items.

In conclusion, the current model provides a novel approach to characterizing BD based on a strong statistical model. This model combines salient items with strong clinical validity to offer an objective pattern of BD consumption, independent of consumption cut-offs and population type, that is applicable regardless of gender. We now have a useful instrument to assess each participant’s BD severity [Appendix and online access (see footnote 1 and 2)] or to identify 4 homogeneous groups of alcohol drinking in a whole sample and differentiate types of drinking, including BD and high-intensity BD with a gradual severity [Appendix and online access (see footnote 2)]. With a simple to use model, the BD severity of each subject, male or female, can be determined according to a global definition that includes consumption, behavioral, and physiological criteria.

## Data availability statement

The raw data supporting the conclusions of this article will be made available by the authors, without undue reservation.

## Ethics statement

The studies involving human participants were reviewed and approved by Comité de Protection des Personnes Nord-Ouest II and Comité de Protection des Personnes Est I. The patients/participants provided their written informed consent to participate in this study.

## Author contributions

JA and MN developed the study. JA, MN, MM, and MD contributed to the study design. JA and FG conducted the data collection and data analyses were performed in collaboration with JA, MN, MM, MD, and OO. JA drafted the manuscript in collaboration with MN, MM, and MD. OP provided critical revisions. FF provided the website. All authors approved the final versions of the manuscript.

## Funding

The study has been supported by the INCa national institute of cancer and IReSP research institute on public health, grant N° INCa/IReSP 16134. The present study was supported by the INSERM and the University of Picardie Jules Verne.

## Conflict of interest

The authors declare that the research was conducted in the absence of any commercial or financial relationships that could be construed as a potential conflict of interest.

## Publisher’s note

All claims expressed in this article are solely those of the authors and do not necessarily represent those of their affiliated organizations, or those of the publisher, the editors and the reviewers. Any product that may be evaluated in this article, or claim that may be made by its manufacturer, is not guaranteed or endorsed by the publisher.

## Footnotes

^1^https://extra.u-picardie.fr/bdct/macro.html

^2^https://extra.u-picardie.fr/bdct/

## Appendix

*(A) To characterize consumption for a whole population.*

A,1: Excel file online access: https://extra.u-picardie.fr/bdct/macro/

A,2: R-code

##six_drink_frq=0 (never); six_drink_frq=1 (<once/month);six_drink_frq=2 (once/month);six_drink_frq=3 (once/week);six_drink_frq=3 (almost every day)

###cons_speed in number of drinks per week

##drunkness_freq (number of times drunk in the last 6 months)

##drunkness_perc (de 0 ‡ 10)

##hang_perc (de 0 ‡ 10)

Bing_category=function(six_drink_freq,cons_speed,drunkness_freq,drunkness_perc,hang_perc)

{

P_sup2=exp(-5.87+0.43*six_drink_freq+0.20*cons_speed+0.34*drunkness_freq+0.68*drunkness_perc+0.36*hang_perc)/(1+exp(-5.87+0.43*six_drink_freq+0.20*cons_speed+0.34*drunkness_freq+0.68*drunkness_perc+0.36*hang_perc))

P_sup3=exp(-7.37+0.43*six_drink_freq+0.07*cons_speed+0.34*drunkness_freq+0.11*drunkness_perc+0.05*hang_perc)/(1+exp(-7.37+0.43*six_drink_freq+0.07*cons_speed+0.34*drunkness_freq+0.11*drunkness_perc+0.05*hang_perc))

P4=exp(-8.46+0.43*six_drink_freq-0.12*cons_speed+0.34*drunkness_freq+0.02*drunkness_perc-0.34*hang_perc)/(1+exp(-8.46+0.43*six_drink_freq-0.12*cons_speed+0.34*drunkness_freq+0.02*drunkness_perc-0.34*hang_perc))

return(c(1-P_sup2,P_sup2-P_sup3,P_sup3-P4,P4))

}

### if six_drink_frq=0; cons_speed=2; drunkness_freq=1; drunkness_perc=2 (20%) and hang_perc=3 (30%)

Bing_category(0,2,1,2,3)

*Note: The PPOM algorithm R-code categorizes the consumption of the whole population by providing the probability of belonging to one of the four group for each subject.*

*(B) The 5 items tool to characterize the consumption of one participant.*

**Online access:** https://home.mis.u-picardie.fr/~furst/bdbq.html

This questionnaire evaluates your **Binge Drinking** habits. Binge Drinking is a massive, occasional and repeated alcohol consumption to get drunk.

Please select the answer that fits you best for each of these 5 questions

**1- How often do you have six or more drinks on one occasion?**

*Never                 0*

*Less than once in a month        1*

*Once in a month              2*

*Once in a week             3*

*Almost every day             4*

**2- When you drink, how fast do you drink?**

*1 drink in 3 hours           1/3*

*1 drink in 2 hours           1/2*

*1 drink in 1 hour            1*

*2 drinks per hour              2*

*…                   …*

*+ 20 drinks per hour.            20*

**3- How many times have you been drunk in the past 6 months?**

Being drunk involves loss of coordination, nausea and/or inability to speak clearly.

*Never                 0*

*…                  …*

*50 times                 50*

**4- In 10 drinking occasions, how many times have you been drunk after drinking?**

*Never                 0*

*1 time out of 10           0,1*

*….                 …*

*10 times out of 10             10*

**5- In 10 drinking occasions, how many times have you had a «hangover» after drinking?**

*Never                 0*

*1 time out of 10           0,1*

*….                …*

*10 times out of 10            10*

*The BDQ score is calculated with the PPOM algorithm.*

*Note: The probability of belonging to one of the four groups for each participant is calculated with the PPOM algorithm.*
